# Suicide probability among physicians: an explainable machine learning analysis of depression, burnout, anxiety, and coping styles

**DOI:** 10.3389/fmed.2026.1943490

**Published:** 2026-09-22

**Authors:** Figen Ünal Demir, Esma Akpınar Aslan

**Affiliations:** Department of Psychiatry, School of Medicine, Tokat Gaziosmanpaşa University, Tokat, Türkiye

**Keywords:** anxiety, burnout, depression, explainable AI, machine learning, physicians in Turkey, suicide

## Abstract

**Objective:**

Suicide is a major public health concern, and physicians are at increased risk because of demanding working conditions and a high prevalence of mental health problems. This study aimed to investigate the sociodemographic characteristics, depression, anxiety, burnout, coping styles, and suicide probability among physicians in Türkiye and to develop and interpret an explainable machine learning model for predicting suicide probability alongside conventional statistical analyses.

**Methods:**

In this cross-sectional study, 769 actively practicing physicians completed a sociodemographic questionnaire, the Beck Depression Inventory (BDI), Beck Anxiety Inventory (BAI), Suicide Probability Scale (SPS), Maslach Burnout Inventory (MBI), and Ways of Coping Inventory (WCI). Significant predictors of SPS scores were identified using multiple linear regression. An Extreme Gradient Boosting (XGBoost) model was subsequently developed, and SHapley Additive exPlanations (SHAP) were applied to interpret model predictions and quantify the contribution of individual predictors.

**Results:**

Multiple linear regression identified BDI, BAI, MBI-depersonalization, MBI-reduced personal accomplishment, WCI-optimistic approach, WCI-helpless approach, and WCI-seeking social support as significant predictors of SPS scores. The XGBoost model demonstrated good predictive performance, comparable to that of linear regression; SHAP analysis showed a broadly concordant pattern of feature contributions to the model's predictions, with depressive symptoms emerging as by far the most influential contributor to suicide probability.

**Conclusions:**

The concordance between conventional regression and explainable machine-learning findings indicates that the two analytical approaches converge on a similar pattern of associations between depression, anxiety, burnout, coping style, and suicide probability; in this study, XGBoost did not provide a substantial predictive advantage over conventional regression, and its principal contribution was complementary model interpretation through global and individual-level SHAP explanations. Given the single-sample, single-split design of the present analysis, these explainable artificial intelligence findings should be regarded as hypothesis-generating rather than as a validated basis for clinical risk prediction, pending external validation.

## Introduction

There has been increasing interest in physicians’ mental health and suicide in recent years. Studies indicate that being a medical doctor is associated with an increased risk of burnout, depression, anxiety disorder, and suicide (1, 2) Factors such as excessive or conflicting job demands, imbalances between work and family life, limited resources, restricted autonomy, fear of making mistakes, long working hours, and interpersonal conflicts are significant in explaining changes in physicians’ mental health (1–3).

Similar to the general population, depression and anxiety disorders are the most common psychiatric conditions among physicians. In two meta-analyses conducted in physicians, anxiety rates in physicians were 26.3% and 25.8%, and depression rates were 28.4% and 20.5% (4, 5). However, despite these high rates of psychiatric distress, physicians’ rates of seeking psychiatric help are significantly lower than expected due to concerns such as potential negative impacts on their career, confidentiality breaches, discomfort with consulting a colleague, denial of psychiatric illness, time constraints, and financial barriers (6–8). However, untreated mental illnesses are associated with an increased risk of suicide. A study by Menon et al. investigating the relationship between depression, burnout, medical errors, and suicide among physicians found that an increase in depression scores was associated with a high increase in suicidal ideation (9).

The term suicide encompasses a spectrum ranging from wanting to die, suicidal ideation, suicide plans, suicide attempts, to death by suicide (10). Suicide ideation, defined as thoughts or wanting to end one's life, is associated with suicide attempts (11). Suicide is a global health problem, with approximately 700,000 deaths by suicide annually (12). According to a 2020 study in the United States, 23% of male physicians and 22% of female physicians reported experiencing suicidal ideation within the past year, with 1% and 2%, respectively, attempting suicide (13). A nationwide registry-based study in Denmark found that physicians had a higher suicide risk than any of 55 other occupations, with this estimated risk further increasing after adjusting for sociodemographic factors (14). Increased suicide rates persist among physicians compared to the general population, especially among female physicians (15, 16). Among physicians themselves, certain specialties, such as anesthesiology, public health, family medicine, and psychiatry, are reported to have higher suicide rates (17). A study examining 61 physician suicides in Türkiye between 2011 and 2021 identified the highest suicide rates among cardiovascular surgeons, family physicians, and obstetricians/gynecologists (18). Another study by Unler and Ertek, analyzing 138 physician suicides in Türkiye through social media and news reports between 2006 and 2021, revealed that most cases were specialists (39.9%), with the most common methods being drug overdose (27.9%) and jumping from heights (21.7%). The specialties with the highest rates of suicide included anesthesiology (12.5%), obstetrics/gynecology (10.2%), and psychiatry (10.2%). Family issues (26.5%), followed by professional/academic problems (22.1%) were cited as the most frequent reasons for suicide (19). More recently, Eskin et al. reported that, during the last month, 9.6% of physicians had experienced suicidal ideation, 3.1% had made a suicide plan, and 1.2% had attempted suicide. The study further demonstrated that negative life events and psychological distress were significantly associated with suicidal behavior among physicians (20). These findings further highlight the considerable psychosocial burden associated with the medical profession. Within the context of these stressful life events, ways of coping may also influence suicidal behavior among physicians. Studies have shown that ways of coping are associated with suicide ideation and suicide attempts (21, 22).

Burnout, another common mental health condition among physicians, is also associated with physician suicides (3). Burnout syndrome, characterized by emotional exhaustion, depersonalization, and reduced personal accomplishment, often results from excessive workplace stress (23). Systemic factors such as excessive workload, the threat of malpractice lawsuits, long working hours, frequent on-call duties at night or on weekends, the obligation to continue working at home, the use of electronic health records and computer-registered data entry, or perfectionism, gender, whether or not to have children, and stress coping strategies play a role in physician burnout (3, 24). Studies found that one-third to two-thirds of physicians experience burnout (25, 26). Burnout has important consequences not only for physicians’ well-being but also for patient safety, healthcare quality, workforce sustainability, and healthcare costs (3).

Although these psychological and occupational factors have consistently been associated with suicide risk among physicians, previous studies have predominantly relied on conventional statistical approaches, which may not fully capture the complex and potentially nonlinear relationships among multiple interacting risk factors.

Recent advances in machine learning have demonstrated promising performance in predicting mental health outcomes and suicide risk by modeling complex nonlinear interactions among demographic, psychological, and clinical variables (27, 28). Nevertheless, many machine learning algorithms remain difficult to interpret because they operate as “black-box” models, limiting their clinical applicability and reducing clinicians’ confidence in model predictions (29). Explainable artificial intelligence (XAI) addresses this limitation by providing transparent and interpretable explanations of model predictions, thereby enhancing model transparency, facilitating clinical trust, and supporting decision-making (29). Recent studies have highlighted the growing value of explainable machine learning in psychiatric research and digital mental health applications (29, 30)*.* To the best of our knowledge, studies integrating conventional psychiatric epidemiological analyses with explainable machine learning to investigate suicide risk among physicians remain scarce. Consequently, the potential clinical utility of explainable artificial intelligence for physician suicide risk assessment has not yet been adequately explored.

This study aimed to investigate the sociodemographic characteristics, depression, anxiety, burnout, coping styles, and suicide probability among physicians in Türkiye. Furthermore, we sought to develop and interpret an explainable machine learning model to complement conventional statistical analyses, evaluate the robustness of the identified predictors, and improve the transparency of suicide risk prediction.

## Method

### Sampling

A total of 769 medical school graduate physicians participated in the study. Individuals experiencing episodes of psychotic or bipolar disorders were excluded from the study. Physicians aged between 23 and 65 who were actively working were included in the study. The sample size was calculated based on the 22.3% prevalence of suicidal behavior reported by Eskin et al. (2024) among physicians in Türkiye (20). Assuming a 95% confidence level and a 5% absolute margin of error, the minimum required sample size was calculated as 266 participants. To account for potential data loss and to support multivariable analyses, recruitment beyond this minimum was targeted. The final sample comprised 769 physicians.

### Procedure

Ethical approval for the research was obtained from the Tokat Gaziosmanpaşa University Clinical Research Ethics Committee (approval date: October 12, 2023; number: 83116987-639) (Clinical trial number: not applicable). This study was conducted in accordance with the Declaration of Helsinki. Physicians actively working were invited to participate in the study via convenience sampling through various social media platforms from November 2023 to February 2024. Sociodemographic data form, Beck Depression Inventory (BDI), Beck Anxiety Inventory (BAI), Suicide Probability Scale (SPS), Maslach Burnout Inventory (MBI), and Ways of Coping Inventory (WCI) are included in the electronic form respectively. Each email address is allowed only one login. Informed consent was obtained from all participants.

### Explainable artificial intelligence (XAI) analysis

In addition to the conventional regression-based approach, an explainable machine learning model was developed to further examine the associations between the psychological and coping variables and suicide probability. An Extreme Gradient Boosting (XGBoost) regression algorithm was trained to predict SPS total scores using the same ten independent variables entered into the linear regression model (BDI, BAI, and the three MBI and five WCI sub-scale scores). The sample was randomly divided into a training set (80%, *n* = 615) and a held-out test set (20%, *n* = 154). Hyperparameters (number of estimators, maximum tree depth, learning rate, subsample ratio, and column subsample ratio) were optimized by grid search with 10-fold cross-validation on the training set, and the final model was subsequently evaluated on the independent test set, which was not used during training or hyperparameter tuning. Model performance was assessed using the coefficient of determination (R^2^), mean absolute error (MAE), and root mean square error (RMSE). To interpret the contribution of each variable to the model's predictions at both the population and individual level, SHapley Additive exPlanations (SHAP) values were computed using the TreeExplainer algorithm (31). SHAP values, derived from cooperative game theory, quantify the marginal contribution of each feature to a given prediction relative to the average model output, providing a transparent and mathematically grounded account of variable importance and directionality that complements the standardized beta coefficients obtained from linear regression. All machine learning analyses were performed in Python (version 3.11) using the XGBoost and SHAP libraries.

The ten psychometric predictors entered into the XGBoost model were identical to those used in the linear regression model; this *a priori* restriction was a deliberate design choice made to preserve direct, like-for-like comparability between the conventional regression and machine-learning analyses, and it is acknowledged that the model was consequently not permitted to select sociodemographic or occupational variables, nor to identify predictor combinations, beyond those already specified by the regression framework. To benchmark the predictive performance of XGBoost against alternative algorithmic families, five additional regression models—ordinary least-squares linear regression, elastic net regression, support vector regression with a radial basis function kernel, random forest regression, and a multilayer perceptron—were trained on the identical ten predictors, using the same 80%/20% training-test partition and the same held-out test set (*n* = 154) as the primary XGBoost model, so that all six models were evaluated under strictly comparable conditions. For each comparator model, hyperparameters were optimized by grid or randomized search with the same 10-fold cross-validation scheme applied to the training set.

To assess whether the exclusion of sociodemographic and occupational characteristics materially affected the findings, a sensitivity analysis was conducted in participants with complete data for the additional covariates (*n* = 713). The original ten psychometric predictors were retained, and 16 available sociodemographic and occupational variables were additionally incorporated into hierarchical linear regression and XGBoost models. For direct comparison, the psychometric-only and expanded models were evaluated within the same complete-case sample; the XGBoost models used identical training and held-out test observations, with hyperparameter optimisation restricted to the training data. SHAP values and exploratory pairwise SHAP interaction values were additionally examined in the expanded XGBoost model.

For the primary XGBoost model, an exhaustive grid search was performed over the following discrete hyperparameter values (324 combinations in total): number of estimators ∈ {100, 200, 300}; maximum tree depth ∈ {2, 3, 4, 5}; learning rate ∈ {0.01, 0.05, 0.10}; subsample ratio ∈ {0.7, 0.8, 1.0}; and column-subsample ratio ∈ {0.7, 0.8, 1.0}, evaluated with 10-fold cross-validation on the training set using the coefficient of determination (R^2^) as the scoring criterion. The combination maximizing the mean cross-validated R^2^ was n_estimators=100, max_depth=3, learning_rate=0.05, subsample=0.8, and colsample_bytree=0.7 (mean cross-validated R^2^ = 0.710), with a fixed random seed of 42 applied throughout data partitioning, cross-validation, and model fitting to ensure computational reproducibility; the identical optimum was independently confirmed via a randomized search of the same space, supporting the stability of this result. The five comparator models were each tuned by grid search with the same 10-fold cross-validation scheme and R^2^ scoring criterion, over the following search spaces: elastic net, regularization strength *α* ∈ {0.01, 0.1, 0.5, 1, 5} and L1 ratio ∈ {0.1, 0.5, 0.9} (selected: *α*=0.1, L1 ratio=0.9); support vector regression (radial basis function kernel), C ∈ {1, 10, 50} and *ε* ∈ {0.1, 0.5, 1} (selected: C = 10, *ε*=1); random forest regression, number of trees ∈ {200, 400}, maximum depth ∈ {3, 5, unrestricted}, and minimum leaf size ∈ {1, 3, 5} (selected: 400 trees, unrestricted depth, minimum leaf size=3); and multilayer perceptron, hidden-layer architecture ∈ {(32), (64), (32,16)} and L2 regularization *α* ∈ {0.0001, 0.001, 0.01} (selected: single layer of 32 units, *α*=0.0001); ordinary least-squares linear regression has no tunable hyperparameters. Predictors were standardized (zero mean, unit variance) prior to fitting for the elastic net, support vector regression, and multilayer perceptron, using scaling parameters estimated from the training set only. As a secondary, exploratory analysis, the continuous XGBoost predictions were additionally evaluated against the conventional SPS≥110 cut-off to derive threshold-based classification metrics (sensitivity, specificity, positive and negative predictive values, F1 score, balanced accuracy, and receiver operating characteristic and precision-recall areas under the curve); given the low prevalence of high-risk cases in the sample (approximately 1.2%), these estimates were expected *a priori* to be statistically unstable, and this analysis was therefore not intended, and should not be interpreted, as a validated clinical classifier. This methodological description addresses applicable items of the TRIPOD (Transparent Reporting of a multivariable prediction model for Individual Prognosis Or Diagnosis) reporting guideline for prediction-model studies, without constituting a formal claim of full TRIPOD compliance.

## Data collection instruments

### Demographic data form

The demographic data form prepared by the researchers includes questions such as participants’ age, gender, marital status, number of children, specialty, years of professional experience, and whether they studied medical school by choice, among others.

#### Beck depression inventory (BDI)

It was developed to determine individuals’ levels of depression (32). This form, which aims to objectively determine the degree of depression, consists of 21 questions and each question is rated between 0 and 3 points. The Turkish version of the scale was validated by Hisli, who determined that a cutoff score of 17 or above had over 90% sensitivity for predicting depression requiring treatment (33).

#### Beck anxiety inventory (BAI)

It was developed to determine subjective anxiety level and physical symptoms, consists of 21 items and each answer is scored between 0−3 (34). The validity and reliability studies of BAI for Türkiye were conducted by Ulusoy, Şahin, and Erkmen, who identified a score of 16 or higher as significant (35).

#### Suicide probability scale (SPS)

SPS, developed to assess suicide risk in adolescents and adults, consists of 36 self-report items (36). The scale has four sub-dimensions: hopelessness (12 items), suicide ideation (8 items), negative self-evaluation (9 items), and hostility (7 items). Higher scores indicate a greater probability of suicide. The Turkish version of the scale was used in this study and the cutoff score is 110 (37).

#### Maslach burnout inventory (mbI)

It was developed by Maslach and Jackson in 1981 (23). This inventory was adapted into Turkish by Ergin in 1993. MBI evaluates burnout in three sub-dimensions: emotional exhaustion, depersonalization, and reduced personal accomplishment. It consists of 22 items in total. Emotional exhaustion scores of 22–30 indicate moderate levels, while scores of 30 and above indicate high levels of burnout. Depersonalization scores of 10–12 indicate moderate levels, while scores of 13 and above indicate high levels of burnout. Reduced personal accomplishment scores of 24–26 indicate moderate levels, while scores of 23 and below indicate high levels of burnout (38).

#### Ways of coping inventory (WCI)

The Turkish validity and reliability of the scale developed by Folkman and Lazarus (1980) was conducted by Şahin and Durak (1995) (39, 40). The scale consists of 30 items and five sub-scales: self-confident approach, optimistic approach, seeking social support, helpless approach, and submissive approach. A higher score on a sub-scale indicates that the corresponding way of coping is used more frequently.

### Data analysis

All statistical analyses were conducted using SPSS Statistics for Windows, Version 22.0 (IBM Corp, 2013). The demographic data of the participants were analyzed with descriptive statistics and reported as mean (standard deviation) or frequency (percentage), and, given the non-normal distribution of several variables, median and interquartile range are additionally reported (see Results). Normality assessment of the variables was performed using skewness and kurtosis values. According to Kim (2013), absolute skewness values >2 and absolute kurtosis values >7 were considered indicative of substantial deviations from normality (41). Since the distributional characteristics of the study variables remained within these acceptable limits and the sample size was large (*n* = 769), parametric methods were considered appropriate. Intergroup differences in quantitative variables were evaluated using the Independent Samples T-Test or One-Way Analysis of Variance (ANOVA); for ANOVA comparisons, homogeneity of variance was assessed with Levene's test, and *post-hoc* pairwise comparisons (denoted by superscript letters in Table 3) were conducted using Tukey's HSD test when variances were homogeneous and the Games-Howell test when Levene's test indicated heterogeneity of variance. Because multiple exploratory comparisons were performed in both subgroup analyses and correlation analyses, no formal correction for multiple testing was applied. Therefore, these findings should be interpreted with caution and considered hypothesis-generating. The reliability of the scales was determined by calculating Cronbach's alpha values for each scale and subscale using item-level responses. Missing item-level responses underlying the three MBI subscales (affecting 28, 60, and 26 participants, respectively, each below 8% of the sample) were handled using mean substitution, allowing the full analytic sample of 769 participants to be retained for both the conventional regression and machine-learning analyses. Two implausible weekly working-hour values (≥176 h/week) were treated as data-entry errors and excluded from analyses involving working hours. Pearson Correlation Analysis and Linear Regression Analysis were performed to examine the relationships between variables. A *p*-value of <0.05 was considered statistically significant.

## Results

A total of 769 actively practicing physicians were included in the study. The participants’ mean age was 37.0 ± 8.34 years, 488 (63.5%) were female, and the mean weekly working time was 42.02 ± 12.65 h. Years of professional experience averaged 12.33 ± 8.46 years.

Because several variables showed non-normal distributions, median and interquartile ranges (IQR) were additionally calculated. The median (IQR) age was 35 (11) years (range, 24–64 years), the median (IQR) professional experience was 11 (12) years (range, 1–39 years), and the median (IQR) weekly working time was 40 (10) hours. Two reported values of ≥176 working hours per week, which were considered physically implausible within a single week, were treated as data-entry errors and excluded from all working-hours calculations. Additional sociodemographic and occupational characteristics of the participants are presented in Table 1.

**Table 1:** Sociodemographic and occupational characteristics of the participants (*n* = 769).

Characteristic	Category	N	%
Gender	Female	488	63.5
	Male	281	36.5
Marital status	Married	518	67.4
	Divorced	45	5.9
	Single	205	26.7
Number of children	No children	337	43.8
	1 Child	186	24.2
	2 Children	196	25.5
	3 Children and More	50	6.5
Professional title	General Practitioner	145	18.9
	Resident	199	25.9
	Fellow	17	2.2
	Specialist	333	43.3
	Assistant professor	33	4.3
	Associate professor	33	4.3
	Professor	9	1.2
Medical specialty	Psychiatry	36	4.7
	Internal diseases	47	6.1
	Family medicine	94	12.2
	Pediatrics	74	9.6
	Neurology	67	8.7
	Physical therapy	29	3.8
	Anesthesiology	39	5.1
	Child and adolescent psychiatry	38	4.9
	Infectious diseases	3	0.4
	Radiology	11	1.4
	Orthopedics	23	3.0
	General surgery	12	1.6
	Urology	10	1.3
	Ophthalmology	12	1.6
	Otorhinolaryngology	5	0.7
	Microbiology	7	0.9
	Pulmonology	19	2.5
	Public health	6	0.8
	Genetics	6	0.8
	Cardiology	11	1.4
	Anatomy	5	0.7
	Dermatology	4	0.5
	Neonatology	2	0.3
	Pediatric neurology	1	0.1
	Pathology	4	0.5
	Emergency medicine	33	4.3
	Radiation oncology	5	0.7
	Pediatric surgery	3	0.4
	Forensic medicine	4	0.5
	Biochemistry	3	0.4
	Allergy and immunology	2	0.3
	Neurosurgery	5	0.7
	Intensive care medicine	3	0.4
	Surgical oncology	1	0.1
	Obstetrics and gynecology	14	1.8
	Gastroenterology	1	0.1
	Endocrinology	2	0.3
	Hematology	2	0.3
	Rheumatology	4	0.5
	Nephrology	1	0.1
	General practitioner	114	14.8
	Thoracic surgery	3	0.4
	Geriatrics	1	0.1
	Neurophysiology	2	0.3
Department	Internal Sciences	508	66.1
	Surgical Sciences	131	17.1
	Basic Sciences	15	2.0
	General Practitioner	114	14.8
On-call duty	No On-Call Duties	274	35.7
	On-Call Only	150	19.6
	Active Duty Only	276	36.0
	On-Call + Active Duty	67	8.7
Inpatient unit	No	386	50.2
	Yes	383	49.8
Patient follow-up in the intensive care unit	No	527	69.1
	Yes	236	30.9
Have you filed a lawsuit?	No	507	65.9
	Yes	262	34.1
Has a lawsuit been filed against you?	No	602	78.3
	Yes	167	21.7
Did you study medical school by choice?	No	118	15.4
	Yes	648	84.6
If you were to take the university entrance exam again, would you choose medical school?	No	485	63.2
	Yes	282	36.8
Are you satisfied with the specialty you are working in?	No	261	35.0
	Yes	484	65.0
Smoking habit	Never	559	72.7
	1–20 cigarettes	153	19.9
	More than 20 cigarettes	57	7.4
Alcohol use	Never	452	58.9
	Several times a month	238	31.0
	Several times a week	78	10.2
Substance use	Never	747	97.1
	Yes-I quitted	22	2.9
Have you received psychiatric treatment?	No	426	55.5
	Yes	342	44.5
Current treatment	No	622	80.9
	Yes	147	19.1
Have you attempted suicide?	No	733	95.3
	Yes	36	4.7
Method of suicide attempt	Medicine	28	77.8
	Other	8	22.2
Family history of psychiatric disorders	No	610	79.3
	Yes	159	20.7
Chronic disease	No	590	76.7
	Yes	179	23.3

Professional title and department represent distinct classifications; therefore, the number of physicians classified as general practitioners may differ between these categories.

Given the non-normal distribution of several scale scores (e.g., SPS-Suicide Ideation: skewness=1.999, kurtosis=1.513), median and interquartile ranges (IQR) were additionally calculated and are presented in Table 2.

**Table 2:** Descriptive statistics and internal consistency coefficients of the study scales.

Scale	Mean ± SD	Median (IQR)	Min-Max	Cronbach's *α*
BDI	14.74 ± 10.03	13 (12)	0–58	0.918
BAI	13.87 ± 11.77	11 (15)	0–61	0.942
SPS	70.58 ± 14.47	68 (18)	39–126	0.911
SPS-Suicide Ideation	10.95 ± 3.81	10 (4)	8–31	0.803
SPS-Hopelessness	24.82 ± 6.85	24 (9)	12–46	0.820
SPS-Hostility	11.57 ± 3.42	11 (4)	7–26	0.861
SPS-Negative Self-Evaluation	23.24 ± 5.33	23 (8)	9–36	0.880
MBI-Emotional Exhaustion	19.48 ± 8.44	20 (12)	1–36	0.933
MBI-Depersonalization	7.35 ± 4.15	8 (5)	1–20	0.763
MBI-Reduced Personal Accomplishment	18.81 ± 5.37	19 (8)	1–32	0.852
WCI-Self-Confident Approach	11.86 ± 4.37	12 (5)	1–21	0.691
WCI-Optimistic Approach	7.79 ± 3.17	8 (5)	1–15	0.861
WCI-Helpless Approach	9.64 ± 4.39	9 (5)	1–24	0.803
WCI-Submissive Approach	6.28 ± 2.51	6 (3)	1–18	0.906
WCI-Seeking Social Support	7.08 ± 2.20	7 (3)	1–12	0.630

BAI, Beck Anxiety Inventory; BDI, Beck Depression Inventory; MBI, Maslach Burnout Inventory; SPS, Suicide Probability Scale; WCI, Ways of Coping Inventory.

Data on participants’ scale scores are presented in Table 2. Moderate-to-severe depressive symptoms were observed in 35.2% of participants, whereas 37.7% exhibited moderate-to-severe anxiety symptoms. According to the SPS cut-off score, 1.2% of participants were classified as being at significant suicide risk. Moderate or higher burnout levels were identified in 39.6% of participants for emotional exhaustion, 29.6% for depersonalization, and 19.3% for reduced personal accomplishment.

The distribution of descriptive characteristics according to SPS total and sub-scale scores is presented in Table 3. No significant gender differences were found in SPS total scores or the suicide ideation, hostility, and negative self-evaluation sub-scale scores. The hopelessness sub-scale score was significantly higher in women (*p* < 0.001). Resident physicians had higher SPS-suicide ideation sub-scale scores compared to specialists, while SPS-hostility sub-scale scores were significantly higher in resident physicians compared to general practitioners, specialists, and academic physicians (*p* = 0.015, *p* = 0.002, respectively). Participants with a history of psychiatric treatment had significantly higher SPS total scores and scores on the suicide ideation, hopelessness, and hostility sub-scales compared to those without such a history (*p* < 0.001). Those with a prior suicide attempt had significantly higher SPS total scores and scores on the suicide ideation and hopelessness sub-scales compared to those without such a history (*p* = 0.018, *p* < 0.001, *p* = 0.029, respectively).

**Table 3:** Distribution of descriptive characteristics based on the suicide probability scale and subscale scores (*n* = 769).

Characteristic	Category	**Suicide Probability Scale**	***Subscales***			
			Suicide Ideation	Hopelessness	Hostility	Negative Self-Evaluation
Gender	Female	71.22 ± 14.81	11.02 ± 3.74	25.5 ± 6.62	11.73 ± 3.33	22.97 ± 5.21
	Male	69.49 ± 13.83	10.83 ± 3.92	23.65 ± 7.11	11.3 ± 3.57	23.71 ± 5.52
	p	0.111	0.504	<0.001*	0.089	0.063
Marital Status	Married	69.07 ± 13.44^a^	10.57 ± 3.38^a^	24.42 ± 6.57	11.31 ± 3.23^a^	22.77 ± 5.31^a^
	Divorced	75.09 ± 14.82^b^	12.09 ± 4.23^ab^	25.82 ± 7.4	11.84 ± 2.92^ab^	25.33 ± 4.25^b^
	Single	73.29 ± 16.19^b^	11.62 ± 4.52^b^	25.58 ± 7.34	12.13 ± 3.88^b^	23.96 ± 5.43^b^
	p#	<0.001*	<0.001*	0.072	0.012*	<0.001*
Professional Title	General Practitioner	70.62 ± 14.12^ab^	10.66 ± 3.6^ab^	24.85 ± 6.87	11.38 ± 3.46^a^	23.74 ± 4.8
	Resident	73.82 ± 15.37^a^	11.73 ± 4.48^a^	25.92 ± 7.38	12.39 ± 3.86^b^	23.78 ± 5.74
	Fellow	67.18 ± 13.7^ab^	9.88 ± 2.32^ab^	23 ± 6.66	11 ± 3.54^ab^	23.29 ± 5.01
	Specialist	69.4 ± 14^b^	10.75 ± 3.57^b^	24.45 ± 6.57	11.35 ± 3.06^a^	22.85 ± 5.28
	Academic physicians	67.95 ± 13.71^b^	10.56 ± 3.28^ab^	23.95 ± 6.41	10.84 ± 3.28^a^	22.6 ± 5.42
	p#	0.003*	0.015*	0.074	0.002*	0.185
Department	Internal Sciences	70.45 ± 14.5	10.95 ± 3.75	24.89 ± 6.71	11.56 ± 3.36	23.05 ± 5.38
	Surgical Sciences	70.34 ± 14.17	10.94 ± 3.98	24.24 ± 7.39	11.58 ± 3.56	23.59 ± 5.62
	Basic sciences	71.2 ± 14.13	11.47 ± 4.53	25.53 ± 5.64	12.07 ± 3.08	22.13 ± 4.56
	General Practitioner	71.27 ± 14.91	10.85 ± 3.81	25.05 ± 7.05	11.53 ± 3.62	23.84 ± 4.83
	p#	0.948	0.950	0.730	0.953	0.346
Did you study medical school by choice?	No	76.15 ± 15.73	12 ± 4.37	27.28 ± 7.46	12.66 ± 3.81	24.21 ± 4.7
	Yes	69.59 ± 14.03	10.76 ± 3.67	24.4 ± 6.65	11.38 ± 3.31	23.05 ± 5.43
	p	<0.001*	0.004*	<0.001*	<0.001*	0.030*
If you were to take the exam again, would you choose medical school?	No	73.41 ± 14.79	11.53 ± 4.08	26.38 ± 6.69	12.07 ± 3.55	23.44 ± 5.1
	Yes	65.64 ± 12.42	9.94 ± 3.03	22.12 ± 6.27	10.7 ± 3	22.88 ± 5.69
	p	<0.001*	<0.001*	<0.001*	<0.001*	0.162
Are you satisfied with the specialty you are working in?	No	76.37 ± 15.56	11.95 ± 4.63	27.54 ± 7.07	12.75 ± 3.88	24.13 ± 4.94
	Yes	67.49 ± 12.76	10.4 ± 3.16	23.4 ± 6.26	10.94 ± 2.96	22.74 ± 5.52
	p	<0.001*	<0.001*	<0.001*	<0.001*	<0.001*
Have you received psychiatric treatment?	No	67.68 ± 13.27	10.2 ± 3.07	23.36 ± 6.65	11.03 ± 3.23	23.09 ± 5.53
	Yes	74.24 ± 15.07	11.89 ± 4.39	26.68 ± 6.64	12.24 ± 3.54	23.44 ± 5.08
	p	<0.001*	<0.001*	<0.001*	<0.001*	0.360
Current treatment	No	69.98 ± 14.31	10.75 ± 3.62	24.5 ± 6.96	11.41 ± 3.41	23.32 ± 5.4
	Yes	73.14 ± 14.89	11.78 ± 4.43	26.22 ± 6.21	12.25 ± 3.4	22.9 ± 5.06
	p	0.017*	0.010*	0.006*	0.007*	0.385
Have you attempted suicide?	No	70.31 ± 14.35	10.84 ± 3.74	24.71 ± 6.84	11.55 ± 3.37	23.2 ± 5.35
	Yes	76.17 ± 15.82	13.03 ± 4.49	27.25 ± 6.71	11.89 ± 4.35	24 ± 4.92
	p	0.018*	<0.001*	0.029*	0.565	0.383

Academic physicians included assistant professors, associate professors, and professors.

P, Independent Samples T-Test.

#p, One-Way Analysis of Variance (ANOVA).

*p < 0.05.

The relationships between SPS total and sub-scale scores and BDI, BAI, MBI sub-scale scores, and WCI sub-scale scores are presented in Table 4. Pearson correlation analysis revealed significant positive correlations between SPS total scores and BDI, BAI, MBI-emotional exhaustion, MBI-depersonalization, WCI-helpless approach, and WCI-submissive approach scores (all *p* < 0.001). Significant negative correlations were found between SPS total scores and MBI-reduced personal accomplishment, WCI-self-confident approach, WCI-optimistic approach, and WCI-seeking social support scores (all *p* < 0.001). Correlations with SPS subscale scores generally showed similar patterns, although some associations varied in magnitude and statistical significance, as detailed in Table 4.

**Table 4:** Relationship between suicide probability scale and subscale scores with other scale scores (*n* = 769).

Scale	Statistic	SPS	Suicide ideation	Hopelessness	Hostility	Negative self-evaluation
BDI	r	0.789	0.657	0.758	0.661	0.273
	p	<0.001*	<0.001*	<0.001*	<0.001*	<0.001*
BAI	r	0.612	0.509	0.610	0.557	0.156
	p	<0.001*	<0.001*	<0.001*	<0.001*	<0.001*
WCI-self confident Approach	r	−0.486	−0.304	−0.350	−0.314	−0.451
	p	<0.001*	<0.001*	<0.001*	<0.001*	<0.001*
WCI-optimistic approach	r	−0.440	−0.252	−0.312	−0.326	−0.403
	p	<0.001*	<0.001*	<0.001*	<0.001*	<0.001*
WCI-helpless approach	r	0.604	0.504	0.622	0.511	0.151
	p	<0.001*	<0.001*	<0.001*	<0.001*	0.002*
WCI-submissive approach	r	0.155	0.160	0.170	0.142	−0.004
	p	<0.001*	<0.001*	<0.001*	<0.001*	0.906
WCI-seeking social support	r	−0.353	−0.257	−0.235	−0.198	−0.345
	P	<0.001*	<0.001*	<0.001*	<0.001*	<0.001*
MBI-emotional exhaustion	R	0.576	0.410	0.613	0.494	0.167
	P	<0.001*	<0.001*	<0.001*	<0.001*	0.032*
MBI-depersonalization	R	0.433	0.313	0.403	0.459	0.138
	P	<0.001*	<0.001*	<0.001*	<0.001*	0.004*
MBI-reduced personal accomplishment	R	−0.355	−0.237	−0.203	−0.174	−0.421
	P	<0.001*	<0.001*	0.015*	0.018*	<0.001*

BAI, Beck Anxiety Inventory; BDI, Beck Depression Inventory; MBI, Maslach Burnout Inventory; SPS, Suicide Probability Scale; WCI, Ways of Coping Inventory.

*p < 0.05.

The results of linear regression analysis for SPS are presented in Table 5. According to the regression analysis, MBI-depersonalization (*β*=0.099; *p* < 0.001), MBI-reduced personal accomplishment (*β*=−0.095; *p* < 0.001), BDI (*β*=0.457; *p* < 0.001), BAI (*β*=0.082; *p* = 0.003), WCI-optimistic approach (*β*=−0.095; *p* = 0.002), WCI-helpless approach (*β*=0.201; *p* < 0.001), and WCI-seeking social support (*β*=−0.155; *p* < 0.001) scores were found to significantly predict SPS scores (R = 0.848, R^2^ = 0.720, adjusted R^2^ = 0.716, F(10,758) = 194.76, *p* < 0.001, Durbin-Watson=2.161). Variance inflation factors for all ten predictors remained below 3 (Table 5), indicating no meaningful multicollinearity.

**Table 5:** Linear regression analysis for suicide probability scale .

Model	B	Std. error	*β* (Beta)	T	p	Confidence interval (95%)		VIF
						Lower limit	Upper limit	
Constant	64.750	1.787		36.231	<0.001*	61.242	68.259	
MBI-emotional exhaustion	0.072	0.050	0.042	1.445	0.149	−0.026	0.169	2.237
MBI-depersonalization	0.373	0.090	0.099	4.132	<0.001*	0.196	0.551	1.550
MBI-reduced personal Accomplishment	−0.255	0.063	−0.095	−4.035	<0.001*	−0.379	−0.131	1.488
BDI	0.660	0.046	0.457	14.291	<0.001*	0.569	0.751	2.772
BAI	0.101	0.034	0.082	2.965	0.003*	0.034	0.167	2.067
WCI-self-confident approach	0.033	0.108	0.010	0.302	0.763	−0.180	0.246	2.903
WCI-optimistic approach	−0.432	0.139	−0.095	−3.113	0.002*	−0.705	−0.160	2.515
WCI-helpless approach	0.662	0.090	0.201	7.374	<0.001*	0.486	0.838	2.006
WCI-submissive approach	−0.154	0.135	−0.027	−1.142	0.254	−0.420	0.111	1.495
WCI-seeking social support	−1.019	0.134	−0.155	−7.597	<0.001*	−1.282	−0.755	1.124
R = 0.848	R2 = 0.720		Adj. R2 = 0.716		Durbin Watson:2.161		F:194.762; *p* < 0.001*	

BAI, Beck Anxiety Inventory; BDI, Beck Depression Inventory; MBI, Maslach Burnout Inventory; WCI, Ways of Coping Inventory.

*p < 0.05.

### Explainable artificial intelligence (XAI) findings

To complement the regression findings and improve the interpretability of the predictive model, the same ten predictors were re-analyzed using an explainable artificial intelligence (XAI) approach. The XGBoost regression model showed stable predictive performance for SPS scores, explaining 71.0% of the variance during 10-fold cross-validation on the training set (R^2^ = 0.710 ± 0.047; MAE=5.98 ± 0.47; RMSE=7.63 ± 0.75) and 67.9% of the variance on the independent held-out test set (R^2^ = 0.679; MAE=6.57; RMSE=8.30), demonstrating stable predictive performance on the held-out internal test set, without implying generalizability beyond this single internal sample and split, and achieved explanatory power comparable to that of the linear regression model evaluated on the same held-out test set (see comparator-model analysis below). SHAP analysis (Figure 1) showed BDI to have by far the largest mean absolute SHAP value among the ten predictors (mean |SHAP|=6.07), i.e., the largest global contribution to the model's predictions, followed by WCI-helpless approach (1.59), WCI-seeking social support (1.39), BAI (1.24), WCI-optimistic approach (0.93), MBI-reduced personal accomplishment (0.90), and MBI-depersonalization (0.79) scores; MBI-emotional exhaustion (0.57), WCI-self-confident approach (0.66), and WCI-submissive approach (0.14) contributed comparatively little to the model's predictions. Consistent with the linear regression results, higher BDI, BAI, MBI-depersonalization, and WCI-helpless approach scores were associated with higher predicted SPS scores, whereas higher MBI-reduced personal accomplishment, WCI-optimistic approach, and WCI-seeking social support scores were associated with lower predicted SPS scores. The concordance between the linear regression and the SHAP-based XAI findings, obtained through two different modeling approaches applied to the same predictors and outcome, indicates that both approaches converge on a broadly similar pattern of associations between depression, anxiety, burnout, and coping style and suicide probability among physicians; this concordance reflects agreement between models rather than independent external validation of these associations.

**Figure 1:**
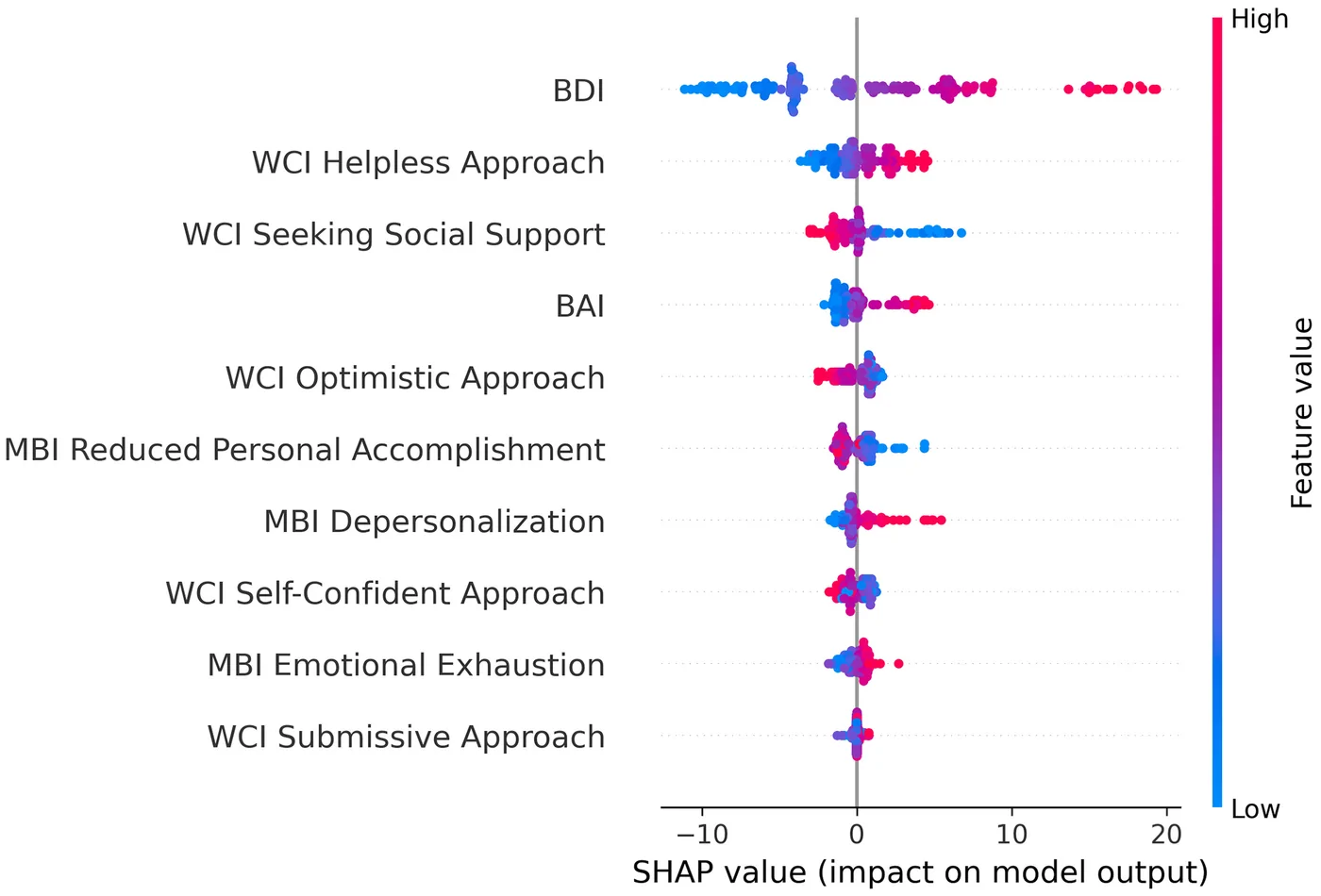
SHAP summary plot showing the impact of each predictor on the XGBoost model's SPS score predictions. The figure was generated from the final XGBoost model (n_estimators=100, max_depth=3, learning_rate=0.05, subsample=0.8, colsample_bytree=0.7, random_state=42) evaluated on the held-out test set (*n* = 154) using TreeExplainer. Each point represents one participant; position on the horizontal axis indicates the contribution of that variable's value to the predicted SPS score (SHAP value), and color indicates whether the participant's value on that variable was high (red) or low (blue). Variables are ordered from top to bottom by mean absolute SHAP value (overall importance). BDI, Beck Depression Inventory; BAI, Beck Anxiety Inventory; MBI, Maslach Burnout Inventory; WCI, Ways of Coping Inventory.

When benchmarked against five alternative algorithms on the identical held-out test set, test-set R^2^ values were 0.659 (MAE=6.76, RMSE=8.56) for linear regression, 0.661 (MAE=6.74, RMSE=8.54) for elastic net regression, 0.611 (MAE=7.03, RMSE=9.13) for support vector regression, 0.664 (MAE=6.65, RMSE=8.49) for random forest regression, and 0.579 (MAE=7.44, RMSE=9.51) for the multilayer perceptron, compared with 0.679 (MAE=6.57, RMSE=8.30) for XGBoost; the corresponding 10-fold cross-validated R^2^ values on the training set were 0.713 ± 0.060, 0.715 ± 0.058, 0.646 ± 0.041, 0.689 ± 0.049, and 0.634 ± 0.069, respectively, compared with 0.710 ± 0.047 (MAE=5.98 ± 0.47, RMSE=7.63 ± 0.75) for XGBoost. XGBoost therefore achieved the highest test-set R^2^ and the lowest test-set error among the six candidate models, but its advantage over the linear and elastic-net baselines was modest, and no nonlinear algorithm clearly or substantially outperformed conventional linear regression. To formally quantify the uncertainty of this comparison, paired bootstrap resampling (5,000 resamples) of the held-out test set was used to estimate 95% confidence intervals for the difference in test-set R^2^ between XGBoost and the two closest-performing linear approaches; the resulting intervals included zero for both comparisons (XGBoost−linear regression: *Δ*R^2^ = 0.019, 95% CI −0.013 to 0.057; XGBoost−elastic net: *Δ*R^2^ = 0.017, 95% CI −0.015 to 0.051), indicating that XGBoost's numerically higher test-set R^2^ should not be interpreted as a statistically robust or practically meaningful predictive advantage over linear modeling approaches. This pattern indicates only that none of the nonlinear algorithms evaluated here captured additional predictive signal beyond conventional linear modeling in this sample; it does not, by itself, establish that the underlying biological or psychometric relationships are truly additive or linear, and should be interpreted accordingly. Sensitivity analyses incorporating the available sociodemographic and occupational variables likewise provided little incremental explanatory or predictive information and did not improve held-out XGBoost performance. The limited SHAP importance and interaction contributions of these variables suggest that they were not major additional sources of predictive signal in the present dataset.

In the secondary, exploratory threshold-based evaluation using the conventional SPS≥110 cut-off, only 3 of the 154 held-out participants (1.9%) met criteria for high suicide-probability risk. Using the continuous XGBoost predictions as the classification score, the model correctly identified 1 of these 3 cases without misclassifying any low-risk participant as high-risk (sensitivity=0.33, specificity=1.00, positive predictive value=1.00, negative predictive value=0.99, F1 score=0.50, balanced accuracy=0.67, ROC-AUC=1.00, PR-AUC=1.00). Because these estimates rest on only three observed high-risk cases in the test set, they are inherently unstable and are reported for completeness only; they should not be interpreted as evidence of validated clinical classification performance, and the continuous SPS score should continue to be regarded as the primary outcome of the predictive model.

Case-level inspection clarified this result: the three observed SPS≥110 cases had predicted scores of 111.5, 106.1, and 103.3, whereas the highest predicted score among all 151 true-negative participants was 102.2—lower than all three positive cases. The model's continuous predictions therefore ranked all three true-positive cases above every true-negative case, yielding perfect rank-based discrimination (ROC-AUC=1.00, PR-AUC=1.00, independently re-verified), even though only one of the three cases crossed the fixed SPS≥110 decision threshold (sensitivity=0.33). This dissociation is mathematically expected, not an artifact: ROC-AUC and PR-AUC evaluate ranking across all possible thresholds, whereas sensitivity is threshold-specific. In other words, near-perfect ranking discrimination did not translate into adequate sensitivity at the prespecified clinical cut-off—an informative and honestly reported limitation of applying a fixed categorical threshold to a continuous prediction derived from only three high-risk observations, rather than evidence of strong classification performance.

To complement the SHAP beeswarm summary plot (Figure 1) and to satisfy the request for additional explainability outputs, four additional SHAP visualizations (Figures 2–5) were generated: a global bar plot of mean absolute SHAP values, a SHAP dependence plot for BDI, and individual-level SHAP waterfall plots for one high-predicted-SPS and one low-predicted-SPS case from the held-out test set. Figures 1–5 were all generated from the single final, reproducible XGBoost model (hyperparameters and random seed as reported in Methods) evaluated on the same held-out test set (*n* = 154).

**Figure 2:**
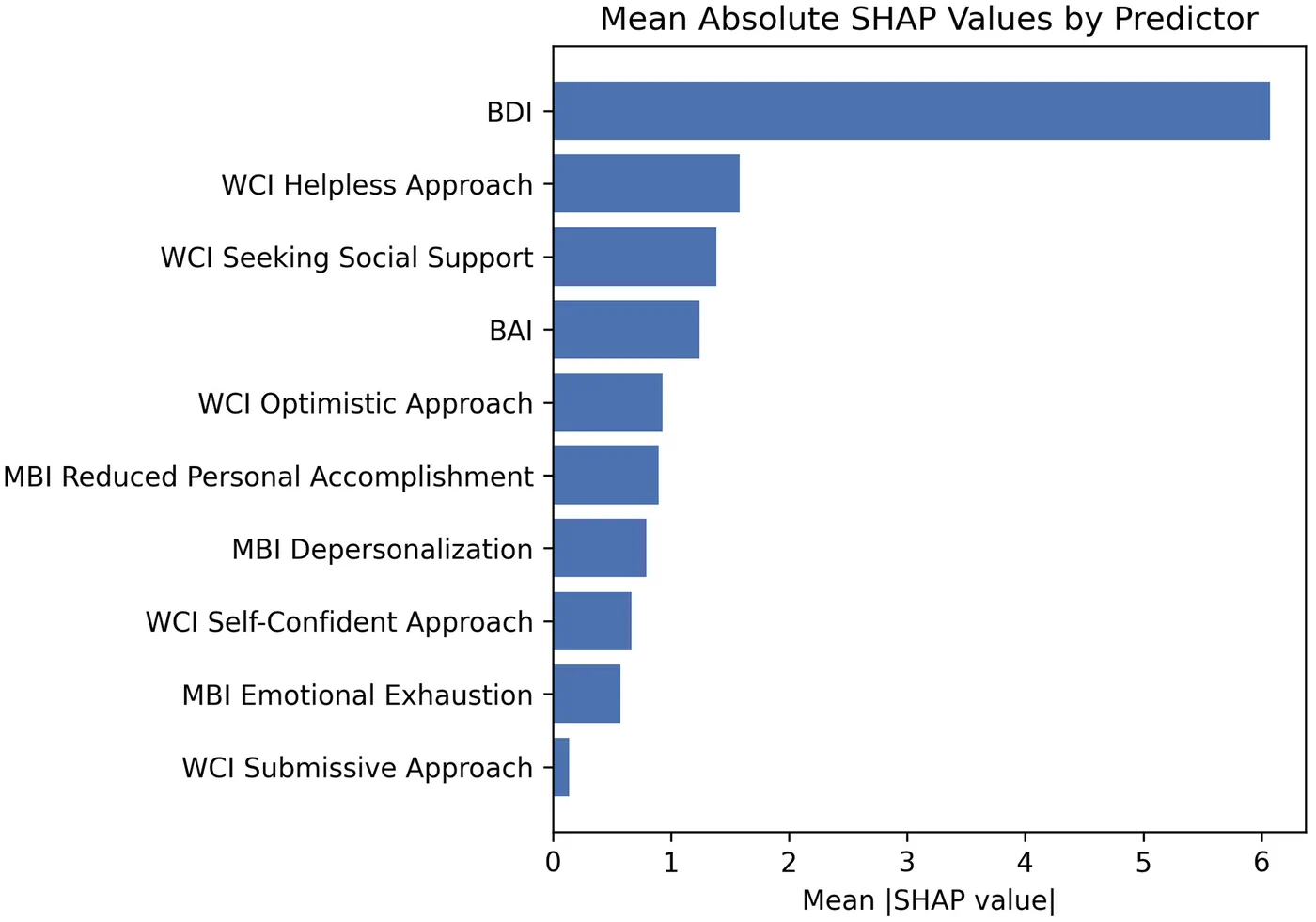
Mean absolute SHAP values for the ten psychometric predictors in the XGBoost model, ranking overall feature importance and complementing the beeswarm plot shown in Figure 1.

**Figure 3:**
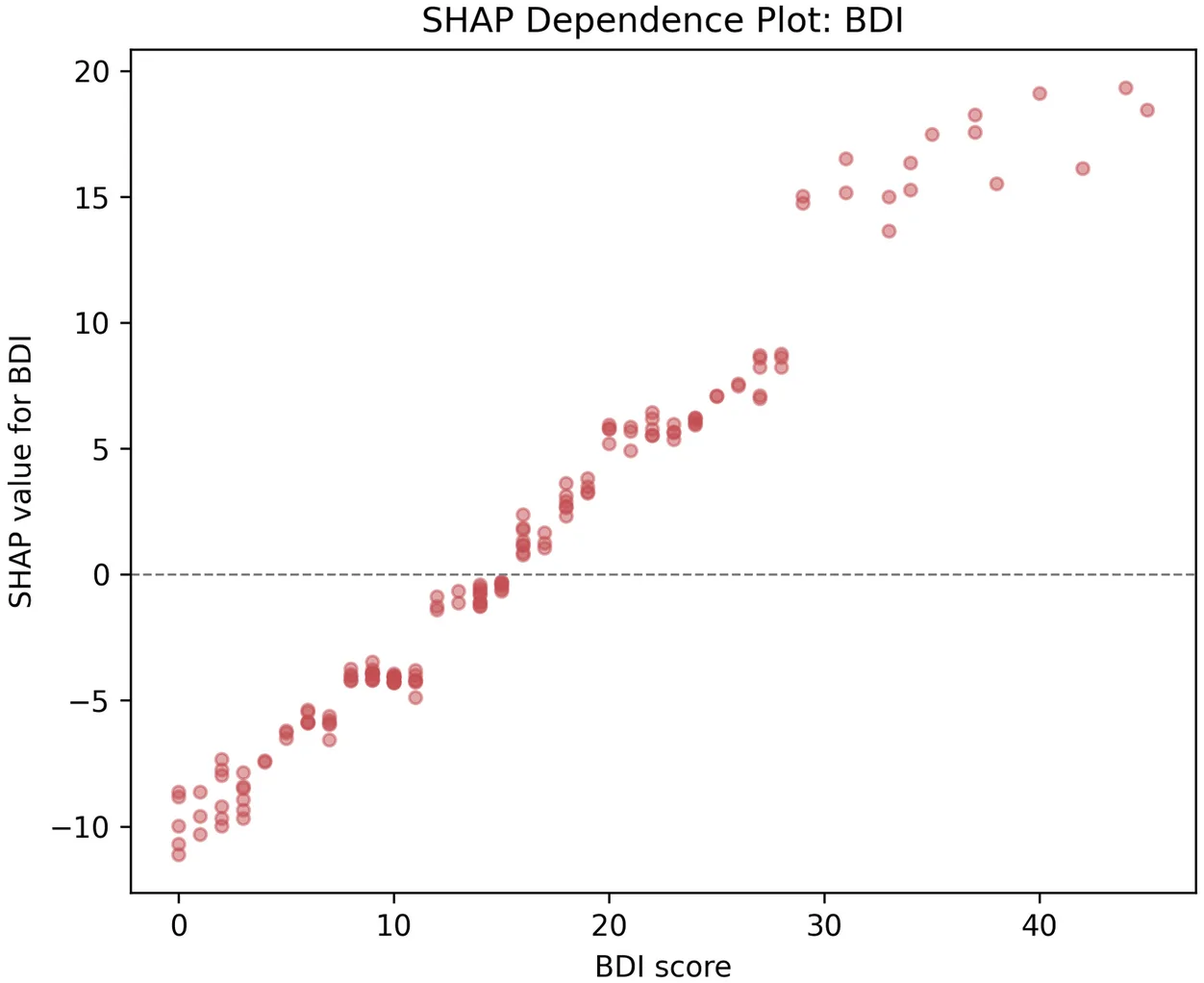
SHAP dependence plot for BDI in the held-out test set, showing the relationship between the BDI score (*x*-axis) and its corresponding SHAP contribution to the predicted SPS score (*y*-axis). The pattern is monotonic, with larger positive SHAP contributions at higher BDI values; this descriptive observation is not a formal test of linearity and should not be taken to establish a specific functional form (e.g., threshold or supralinear) without dedicated nonlinearity testing.

**Figure 4:**
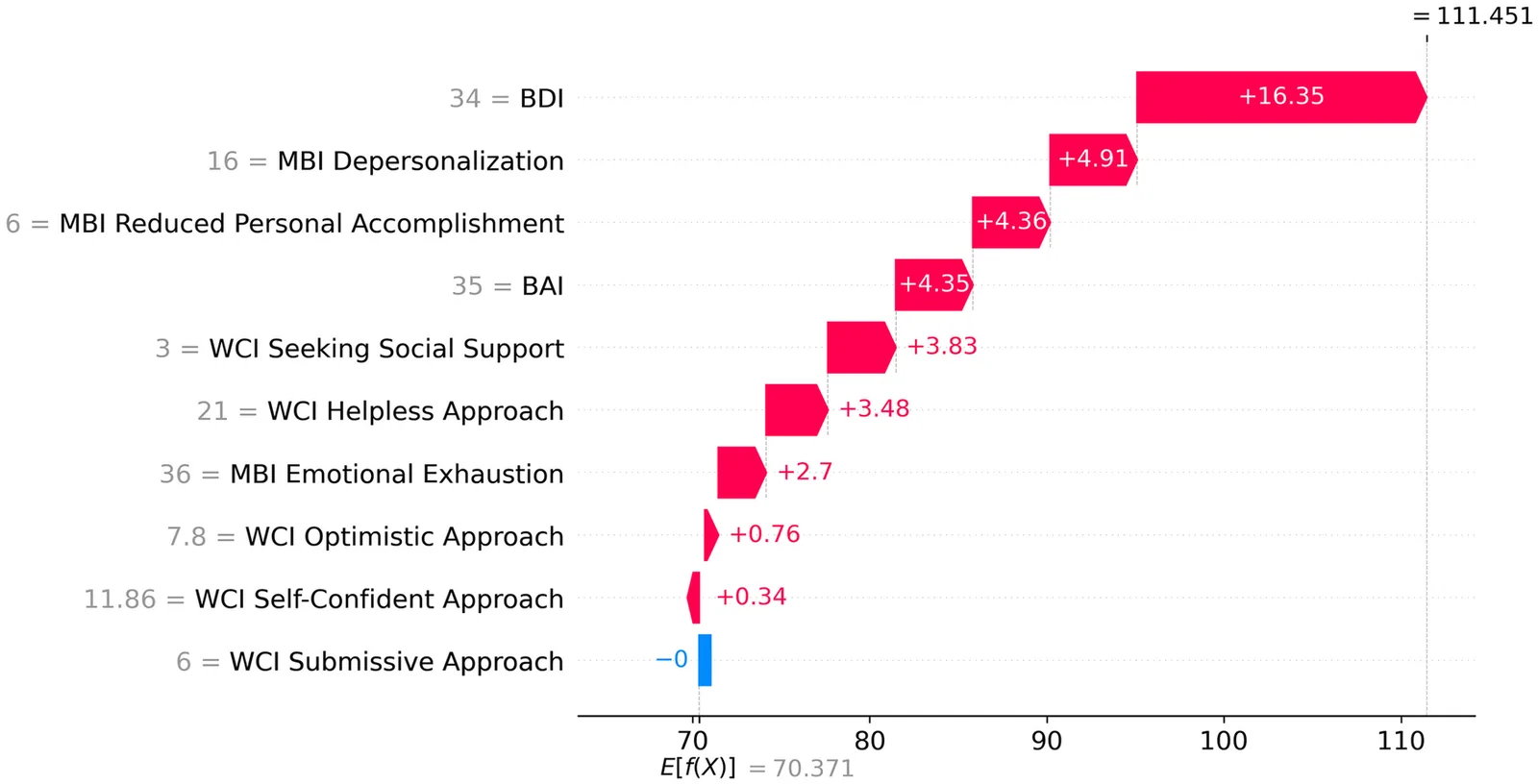
Individual-level SHAP waterfall plot for an illustrative held-out test-set participant with the single highest model-predicted SPS score in the test set (observed SPS=121; predicted SPS=111.5; base value=70.4), selected on this basis rather than by any clinical criterion, illustrating the main positive and negative contributors to this individual prediction.

**Figure 5:**
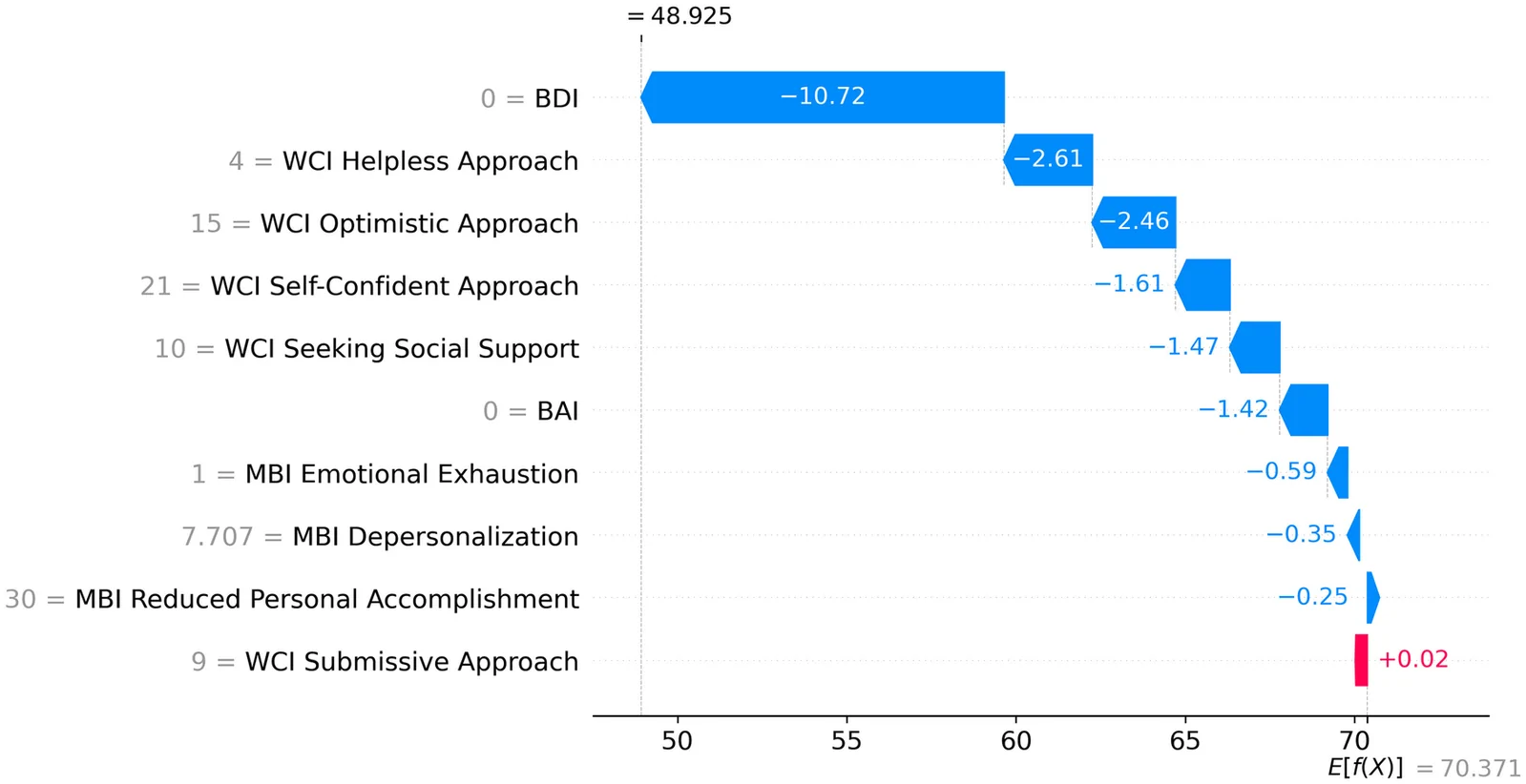
Individual-level SHAP waterfall plot for an illustrative held-out test-set participant with the single lowest model-predicted SPS score in the test set (observed SPS=44; predicted SPS=48.9; base value=70.4), selected on this basis rather than by any clinical criterion, illustrating the main positive and negative contributors to this individual prediction.

As an exploratory extension addressing potential nonlinear interaction effects, pairwise SHAP interaction values were computed for the final XGBoost model on the held-out test set. The five largest mean absolute pairwise interactions were BDI × WCI-seeking social support (0.16), BDI × WCI-helpless approach (0.16), BDI × WCI-optimistic approach (0.16), BDI × BAI (0.16), and WCI-self-confident approach × WCI-seeking social support (0.13); each of these was approximately one order of magnitude smaller than the main effect of BDI (mean |SHAP|=6.59 on the diagonal of the interaction matrix). This indicates that pairwise interactions among the ten predictors contributed only marginally to the model's predictions relative to their additive main effects, and that the predictive contribution of the XGBoost model in this sample derives predominantly from main effects rather than from strong nonlinear interactions; the introductory rationale that machine learning may capture complex nonlinear interactions should therefore be read as a general methodological motivation rather than as a finding demonstrated by the present interaction analysis.

Sensitivity analyses were conducted in the common complete-case sample (*n* = 713). In hierarchical regression, the psychometric-only model explained 72.5% of the variance in SPS scores (R^2^ = 0.725), whereas addition of the sociodemographic and occupational variables increased the explained variance to 73.9% (R^2^ = 0.739). This incremental contribution was small and did not reach statistical significance (*Δ*R^2^ = 0.014, F-change(25,677) = 1.43, *p* = 0.082). In the same sample, adding these variables to XGBoost did not improve held-out predictive performance (R^2^ = 0.694 for the psychometric-only model vs. 0.683 for the expanded model; *Δ*R^2^ = −0.011, 95% CI −0.028 to 0.008). BDI remained the dominant feature in the expanded SHAP analysis. The strongest interaction involving a sociodemographic or occupational variable was between BDI and the number of patients seen per day (mean |SHAP interaction|=0.049), ranking seventeenth among all pairwise interactions. Overall, inclusion of the available sociodemographic and occupational characteristics did not materially alter the principal modelling results.

## Discussion

This study sought to address a gap in the scientific literature regarding suicide risk and associated factors among physicians in Türkiye. To our knowledge, there has not been a large-scale study investigating the relationships between suicide, depression, anxiety, burnout, and sociodemographic data among physicians in Türkiye.

### Depression, anxiety, burnout, and suicidality

In our study, 35.2% of the participants were found to exhibit moderate to severe depressive symptoms, while 37.7% displayed moderate to severe anxiety symptoms. All participants with significant depressive symptoms also exhibited significant levels of anxiety. These rates are notably higher than the prevalence of depression and anxiety in the general population. While our findings align with other studies in the literature, the rates of anxiety and depression among physicians in our study were higher compared to those reported in studies conducted in other countries (4, 5). For instance, in a 2006 study conducted by Erdur et al. on physicians working in emergency departments in Türkiye, the rates of depression and anxiety were found to be 29% and 28%, respectively (42). Akova et al. reported depression and anxiety rates of 37.1% and 27.9% among physicians in their 2021 study (43). The similarity between Akova et al.'s findings and our results underscores consistency with the Turkish literature. The higher anxiety and depression rates observed in our study compared to those in other countries may be associated with the more demanding working conditions for doctors in Türkiye, including heavier patient loads and a higher risk of workplace violence. At the time of the study, 19.1% of the participating physicians were receiving active psychiatric treatment. While BDI and BAI used in this study are screening tools rather than diagnostic instruments, the high rate of participants with moderate to severe symptoms suggests that the rate of those receiving treatment remains relatively low. This may stem from concerns such as avoiding seeking psychiatric help, feeling that the illness is not appropriate for someone in their position, believing they can handle it on their own, worries about confidentiality, the discomfort with consulting a colleague, or fears of their employment contract not being renewed as a result (6, 7).

In our study, moderate or higher levels of emotional exhaustion were found in 39.6% of participants, depersonalization in 29.6%, and reduced personal accomplishment in 19.3%. In the literature, burnout rates among physicians vary between 0% and 89%, influenced by differences in measurement instruments (25, 26). In a study conducted in Japan, the burnout rate was determined to be 34.6% (44). The results of our study are consistent with those reported in studies conducted in other countries. In a study of emergency physicians in Türkiye, emotional exhaustion reached 70%, with higher rates observed among women (45). In a study conducted by Çetin et al. in 2021, the mean scores determined for emotional exhaustion, depersonalization, and reduced personal accomplishment were similar to the results of our study (19.02 ± 6.98, 7.28 ± 3.75, 18.40 ± 3.90) (46). In another study, the mean MBI emotional exhaustion sub-scale score was 20.80 ± 5.96 (range:2–36), mean depersonalization sub-scale score was 10.97 ± 3.92 (range:0–20), and mean reduced personal accomplishment sub-scale score was 18.97 ± 3.89 (range:0–32) (47). The variability in findings may be associated with our study not being conducted on a specific specialty and differences in cutoff points used for the scales. Approximately half of the physicians in our study exhibited burnout symptoms, likely influenced by factors such as increased workload, time constraints, long working hours, interpersonal conflicts, imbalances between work and family life, and individual characteristics. Physician burnout is also associated with adverse outcomes for patient care, physician workforce, healthcare system costs, and physicians’ safety and personal well-being (3).

When the cutoff score for the Suicide Probability Scale (SPS) was set at 110, 1.2% of participants were identified as being at high risk of suicide. The Turkish validity and reliability study of the SPS used in this study was conducted among psychiatric inpatients (77.32 ± 17.87), psychiatric outpatients (71.03 ± 15.85), and healthy controls (60.86 ± 11.13). The mean SPS score in our study was 70.58 ± 14.47, which is closer to the mean score of outpatients in the validation study and higher than that of the control group (37). This finding is consistent with studies reporting higher suicide risk among doctors compared to the general population (14–16, 48).

### Suicidality and descriptive characteristics

Among the 769 physicians in our study, 142 (18.4%) reported having recently thought about suicide, and 36 (4.68%) had attempted suicide in the past. Of the 36 individuals who had attempted suicide, 28 had done so using medications. This finding aligns with reports of higher rates of medication-based suicide attempts among doctors in Türkiye (18). Two meta-analyses identified lifetime suicide attempt prevalence among doctors as 1% and 1.8% (15, 49). In Türkiye, Koparal's study on psychiatrists reported a prevalence of 5.8% for previous suicide attempts (50). The findings of both Koparal's study and ours suggest higher suicide attempt rates among physicians in Türkiye compared to those in other countries, which is likely related to the challenges of being a physician in Türkiye. This is consistent with a recent nationwide study reporting associations between negative life events, psychological distress, and attitudes towards suicide among Turkish physicians (20).

In our study, the hopelessness sub-scale scores for suicide were found to be statistically significantly higher in women than in men. The results of a meta-analysis by Rierra Serra et al. demonstrated that the severity of hopelessness predicts suicide ideation and suicide attempts (51). Another meta-analysis comparing suicides among physicians and non-physicians across 20 countries found higher suicide rates among female physicians compared to non-physician women (52). Similarly, another meta-analysis reported a 1.46 times higher risk of suicide among female physicians (53). The higher levels of hopelessness among female doctors may contribute to increased suicide rates. Factors such as difficulties in balancing family and work life and challenges in establishing a professional presence may also play a role in this disparity (54, 55). Being married is known to be a protective factor against suicide (56). In our study, married participants had lower suicide probability scores compared to both single and divorced individuals. However, while some studies have found a reduced risk among married individuals and a higher suicide risk among divorced individuals compared to singles, our study did not identify a significant difference between singles and divorced individuals (57).

Among resident physicians, both the total score of the Suicide Probability Scale and the sub-scale scores for suicide ideation and hostility were found to be higher compared to specialist physicians. In psychometric measurements conducted by Lopez et al. at the beginning, sixth month, and year-end among resident physicians, a statistically significant increase in anxiety, depression, and suicide risk was observed across all three measurements (58). Similarly, a study conducted in Türkiye investigating the relationship between risky alcohol use, burnout, and suicide among physicians reported significantly higher Suicide Probability Scale scores among resident physicians (59). In our study, due to the large number of subgroups based on participants’ specialties, group comparisons of total SPS scores and sub-scale scores could not be conducted. However, when participants were grouped according to their departments, no significant differences were found between surgical sciences, internal sciences, basic sciences, and general practitioners. This finding is consistent with another study conducted in Türkiye, which also found no differences between departments (59). However, the literature includes studies indicating that suicide risk is higher among anesthesiologists, psychiatrists, general practitioners, and general surgeons (15).

In our study, Suicide Probability Scale scores were found to be significantly higher among physicians who actively worked on night shifts. This finding aligns with a meta-analysis that identified long working hours and shift work as factors associated with increased suicide risk (60). Considering the working conditions of resident physicians in Türkiye, the intense and shift-based workload may contribute to an increased risk of suicidality.

### Suicide and related factors: depression, anxiety, burnout, and ways of coping

Depression and anxiety are significant mental health issues among physicians. The nature of their specialty, inability to afford mistakes, personality traits, and demanding working conditions predispose physicians to mental illnesses. In our study, a significant relationship was found between depression and anxiety scores and suicidality. A study by Menon et al. on physicians showed that a 1-unit increase in depression scores was associated with a 202% increase in the likelihood of suicide ideation (9). A meta-analysis similarly identified hopelessness, BDI scores, and a diagnosis of major depressive disorder as the strongest predictors of suicide ideation (61). Another meta-analysis by Bentley et al. demonstrated that anxiety was a statistically significant but weak predictor of suicide ideation and suicide attempts (62). These findings are consistent with our results.

Another significant finding of our study is the positive correlation between the total SPS score and all its sub-scale scores with the emotional exhaustion and depersonalization sub-scales of MBI, while a negative correlation was found with the reduced personal accomplishment sub-scale score. Additionally, regression analysis revealed that depersonalization and reduced personal accomplishment scores were significant predictors of SPS scores. A study conducted among physicians in Türkiye also found a strong correlation between burnout scores and Suicide Probability Scale scores (59). In a study conducted in Spain, emotional exhaustion among physicians was found to be associated with both active and passive suicide ideation, while reduced personal accomplishment was associated with active suicide ideation (63). Our findings align with the literature. Burnt-out physicians are unlikely to seek professional help and may attempt to cope with substance abuse, depression, and suicidal thoughts on their own (64). Therefore, burnout not only increases suicide risk but also indirectly exacerbates it by hindering help-seeking behavior for accompanying mental health issues. A longitudinal study spanning the COVID-19 pandemic similarly linked burnout and depression to suicidal ideation among physicians and additionally identified perfectionism as a contributing trait-level correlate of suicidal risk (65), an unmeasured construct that the present study was not able to assess but that may partly underlie the burnout-suicide association observed here.

To further validate the findings of the conventional linear regression analysis, we additionally evaluated the predictors of suicide probability using an explainable artificial intelligence (XAI) approach. The XGBoost model demonstrated good predictive performance on the independent test dataset and achieved an explanatory power comparable to that of the linear regression model. The consistency between these two complementary analytical approaches suggests that the associations of depression, anxiety, burnout, and coping styles with suicide probability among physicians are robust and not limited to conventional linear statistical models.

Notably, when XGBoost was benchmarked against a broader panel of regression algorithms spanning linear, regularized-linear, margin-based, ensemble-tree, and neural-network families, it provided only a modest, statistically non-robust improvement in predictive accuracy over the linear and elastic-net baselines (see paired-bootstrap analysis above), and no candidate model achieved a materially higher test-set R^2^ than conventional multiple linear regression. Together with the modest magnitude of the pairwise SHAP interaction values reported below, this finding indicates that none of the nonlinear algorithms evaluated captured a clear predictive advantage over linear modeling in this sample; it tempers, without fully excluding, the narrower interpretation that machine learning was required to capture complex nonlinear interactions among these predictors. Rather, the principal added value of the XGBoost/SHAP framework in this study appears to lie in its capacity to visualize individualized, direction-specific contributions of each predictor at both the population and individual-participant level, which complements, rather than supersedes, the interpretability already afforded by the standardized regression coefficients.

SHAP analysis enabled the interpretation of the model's predictions by quantifying the contribution of each predictor to the estimated suicide probability. In our study, the Beck Depression Inventory (BDI) score emerged as by far the most influential predictor, further emphasizing the central role of depressive symptoms in suicide risk among physicians. In addition, helpless coping, seeking social support, anxiety severity, and burnout dimensions made meaningful contributions to the predictive model. The concordance of the SHAP-based findings with those obtained from linear regression indicates that the two analytical approaches identify a broadly similar pattern of associations across different modeling assumptions; this concordance should be interpreted as internal consistency between models applied to the same sample rather than as independent confirmation or increased statistical reliability of the identified risk factors.

From a clinical perspective, explainable artificial intelligence models may, in principle and pending external validation, serve as valuable clinical decision-support tools for the early identification of physicians at increased risk of suicide. Simultaneous evaluation of depression, anxiety, burnout, and maladaptive coping styles may facilitate earlier recognition of high-risk individuals, particularly among physicians working under demanding conditions. In the present study, however, this potential remains illustrative rather than demonstrated: the model was evaluated on a single internal train-test split with only three high-risk participants in the held-out set, and no external or prospective validation was performed. Such models are intended to support, rather than replace, clinical judgment and should be considered complementary tools within comprehensive psychiatric assessment only after appropriate external validation. Nevertheless, external validation in independent samples, different healthcare systems, and diverse cultural settings is required before explainable artificial intelligence models can be routinely implemented in clinical practice. Furthermore, future studies should evaluate their generalizability and clinical utility while ensuring that these models are developed and applied in accordance with the principles of transparency, fairness, interpretability, and ethical use.

The present reporting addresses applicable items of the TRIPOD (Transparent Reporting of a multivariable prediction model for Individual Prognosis Or Diagnosis) guideline, including explicit specification of the training-test partition, the cross-validation procedure, the final hyperparameters, and the random seed, although full compliance with all TRIPOD items was not formally assessed; prospective external-validation studies are encouraged to adopt this guideline in full.

The last important finding of our study was that there was a positive correlation between the SPS scores and the helpless approach and submissive approach scores, and a negative correlation between the self-confident approach, optimistic approach, and seeking social support scores. The results of the regression analysis indicated that the optimistic approach, helpless approach, and seeking social support scores significantly predicted the SPS scores. This finding is consistent with the result of Bakken et al. in their longitudinal study in adolescents, where the use of less emotional ways of coping were associated with lower suicide ideation (22). In another study conducted with migrant workers, negative ways of coping were associated with suicide risk (66). Results showed that negative coping styles had a positive association with suicide risk. To the best of our knowledge, there is no study in the literature examining ways of coping and suicide risk among physicians. Based on the results of our study, it can be concluded that emotional coping styles are associated with increased suicide risk.

### Strengths and limitations

Several strengths and limitations of the study need to be highlighted. One of the strengths of the current study is the large sample size. Another strength is the use of a multivariate approach, given the complexity of the suicide process. Although the fact that the study was conducted in a single country and that working conditions in Türkiye may influence the results limits the generalizability of the findings, the study provides important data on physician suicide risk in Türkiye and contributes to the limited literature on the subject. However, the study also has certain limitations. The first is that the data were collected online. Since participation was voluntary, it is more likely that individuals with an interest or concern about the research subject completed the survey. Therefore, physicians with a greater interest in or concern about mental health may have been overrepresented in the study, which may have influenced the prevalence estimates in either direction. This can influence the results, particularly in studies conducted on physicians. Another limitation is the cross-sectional nature of the study, which precludes accounting for some influencing factors.

A further limitation is that the primary XGBoost model was restricted *a priori* to the same ten psychometric predictors used in the linear regression to preserve direct comparability between the modelling approaches. Although sensitivity analyses incorporating sociodemographic and occupational variables did not materially change the findings, these analyses were based on a reduced complete-case sample (*n* = 713) and were limited to variables available in the present dataset. Contributions from other demographic, occupational, clinical, or contextual factors therefore cannot be excluded. A recent interpretable-machine-learning study of clinically significant suicide risk in major depressive disorder used sequential-feature selection across a broader candidate predictor set together with *post-hoc* explainability methods (67), and offers a useful template for such an expanded, data-driven approach in future work on physician suicide risk.

## Conclusion

Mental health problems are common among physicians. In addition, challenging working conditions contribute to the mental issues. Therefore, improving working conditions as much as possible and the early recognition of mental disorders are essential. Barriers to seeking help should be well identified, and physicians should be encouraged to seek help when needed. In this study, XGBoost did not provide a substantial predictive advantage over conventional regression; its main contribution was complementary model interpretation through global and individual-level SHAP explanations, which enhanced the transparency of the statistical risk factors already identified by regression, rather than establishing an independently validated basis for clinical risk prediction. Explainable artificial intelligence models of this kind may, pending external and prospective validation, eventually support the identification of physicians at increased risk of suicide and provide additional information to clinicians; however, they should always be used alongside, and never in place of, comprehensive clinical evaluation. Both individual and organizational interventions in this area could contribute to reducing suicide rates.

## Data Availability

The datasets presented in this article are not readily available because the datasets analyzed during the current study are available from the corresponding author on reasonable request. Requests to access the datasets should be directed to funal86@hotmail.com.
